# Solution processes for ultrabroadband and omnidirectional graded-index glass lenses with near-zero reflectivity in high concentration photovoltaics

**DOI:** 10.1038/s41598-018-33200-9

**Published:** 2018-10-08

**Authors:** Junwen He, Yuan Yao, Kyu-Tae Lee, Nina Hong, Brent Fisher, Rabab R. Bahabry, Jung Woo Lee, Jeonghyun Kim, Seungyong Han, Sanjay V. Kalidindi, Jae-Hwan Kim, Sung Bong Kim, Jaewon Choi, Hongwoo Jang, Myeong Namkoong, Scott Burroughs, Muhammad Hussain, Ralph G. Nuzzo, John A. Rogers

**Affiliations:** 1 Department of Chemistry, University of Illinois at Urbana-Champaign, Urbana, IL 61801 USA; 2 Department of Physics, Inha University, Incheon, 22212 Republic of Korea; 3 J. A. Woollam Co., Inc, Lincoln, NE 68508 USA; 4 Semprius, Durham, NC 27713 USA; 5 Department of Physics, Faculty of Science-Al Faisaliah Campus, University of Jeddah, Jeddah, 21589-80200 Saudi Arabia; 6 Department of Materials Science and Engineering, Pusan National University, 2, Busandaehak-ro 63beon-gil, Geumjeong-gu, Busan, 46241 Republic of Korea; 7 Department of Electronics Convergence Engineering, Kwangwoon University, Nowon-gu, Seoul, 01897 Republic of Korea; 8 Department of Mechanical Engineering, Ajou University, San 5, Woncheon-Dong, Yeongtong-Gu, Suwon, 16499 Republic of Korea; 9 Department of Electrical and Computer Engineering, University of Illinois at Urbana-Champaign, Urbana, IL 61801 USA; 10 Department of Materials Science and Engineering and Frederick Seitz Materials Research Laboratory, University of Illinois at Urbana-Champaign, Urbana, IL 61801 USA; 11 Integrated Nanotechnology Lab, CEMSE Division, King Abdullah University of Science and Technology (KAUST), Thuwal, Saudi Arabia

## Abstract

Concentrator photovoltaic (CPV) systems, where incident direct solar radiation is tightly concentrated onto high-efficiency multi-junction solar cells by geometric optical elements, exhibit the highest efficiencies in converting the sun’s energy into electric power. Their energy conversion efficiencies are greatly limited, however, due to Fresnel reflection losses occurring at three air/optics interfaces in the most sophisticated dual-stage CPV platforms. This paper describes a facile one-step wet-etching process to create a nanoporous surface with a graded-index profile on both flat and curved glasses, with capabilities of achieving ~99% average transmission efficiency in a wide wavelength range from 380 nm to 1.3 µm and for a wide range of incident angles up to ±40° regardless of the polarization state of incident sunlight. The simplicity of the etching process remarkably increases their versatility in various optical elements that require unconventional form factors such as Fresnel lenses and microlens arrays, and/or demanding curvatures along with much reduced dimensions such as ball lenses. Etched glass surfaces on two-stage optical concentrating systems yield enhancements in total optical transmission efficiencies by 13.8% and in the photocurrent by 14.3%, as experimentally determined by measurements on microscale triple-junction solar cells. The presented strategy can be widely adapted in a variety of applications such as image sensors, display systems, and other optoelectronic devices.

## Introduction

A recent resurgence of interest in concentrator photovoltaics (CPV) follows from advanced embodiments that increase its potential for use in utility-scale power generation. The most sophisticated systems use geometric optical elements that concentrate solar radiation onto photovoltaic devices with reduced area to allow for the effective use of highly efficient multi-junction (MJ) solar cells. The ability to exploit different semiconductors with multiple bandgaps in such MJ platforms minimizes thermalization and absorption losses as compared to their single-junction flat-plate counterparts. Moreover, advances in epitaxial growth techniques and assembly strategies further improve cell efficiencies, with champion cell efficiencies that now reach 46%^1^. The highest performance CPV systems entail dual-stage concentrating optics, wherein a primary optic focuses incident sunlight onto III-V compound semiconductor based MJ cells with small lateral dimensions, while a secondary optic enhances tracking/alignment tolerance and irradiance uniformity on the cells. Alternative, and sometimes complementary, strategies that employ spectrum splitting optics^2^ and/or diffuse light capture^3^ are also possible. The utilization of state-of-the-art MJ cells in conjunction with such advanced optical concentrating systems enables the most advanced CPV technologies to compete, on a cost basis, with the conventional flat-plate solar cells particularly in geographic regions with strong direct normal irradiance (DNI). Many such CPV systems use optics based on polymers that are highly moldable and cost effective, but their long-term reliability remains questionable because of their vulnerability to degradation in harsh outdoor conditions—notably abrasion, yellowing and mechanical cracking/fatigue. Glass, on the other hand, is a choice material for field deployment, especially for the secondary optics where both optical and mechanical stress are most intense due to the high irradiance levels they are exposed to.

Whether comprised of plastic or glass, the optics in existing CPV platforms lead to substantial Fresnel reflection losses at their interfaces with air due to index mismatch—for example, a ~12% reflection loss arises from 3 air/optic interfaces in the most sophisticated CPV modules, which use two optics stages: a primary Fresnel/plano-convex lens or lens array integrated with a refractive secondary optic based on a dome/ball lens. Antireflection coatings (ARCs) are therefore essential. While reflection losses at interfaces between air and semiconductors with high indices of refraction (e.g., III-V compound semiconductors) can be easily mitigated by utilizing standard multilayer coatings, reducing Fresnel reflections at air/optic interfaces can be much more challenging. One of the simplest and best-known approaches exploits thin-film optical coating methods: (1) a single-layer quarter-wavelength coating that cancels the reflection at a specific wavelength when the refractive index of a medium is between air and the optic according to $\sqrt{{n}_{air}{n}_{optic}}$; and (2) a multi-layer coating comprising alternating dielectric media with varying refractive indices for broad spectral and angular coverages. These two approaches are limited by the absence of materials with appropriate refractive indices (optimum value for a single-layer ARC is *n*~1.2, while the lowest refractive indices for dense dielectrics are on the order of 1.38, such as magnesium fluoride (MgF_2_)). Although strategies based on patterning (via self-assembly^4–6^ and lithographic techniques such as nanoimprint^7,8^) the air/optic interface with subwavelength periodic structures, i.e., forming “moth-eye” surfaces^9,10^ that allow only zeroth-order diffracted rays to transmit and suppresses any higher-order diffractions^11,12^, the fabrication of such subwavelength-scale structures, especially on curved surfaces such as optical lenses, is difficult at costs and volumes for realistic use in CPV. An interesting alternative is to introduce air voids in dense coating materials to achieve the necessary low refractive indices in an averaged sense. According to the effective medium theory^13^, a porosity of 60% will reduce the refractive index of a glass medium (n~1.5) to 1.2. To avoid light scattering, the pores must have homogenous distributions of size in the subwavelength regime^14^. Such nanoporous systems can be realized by phase separation^15,16^, etching^17^, template removal^18^, oblique angle deposition^19–21^ and sol–gel^22^ techniques. Nevertheless, creating such ARCs on primary optics with unconventional form factors and/or secondary optics in CPV systems, the latter of which typically involve high curvatures and small dimensions, represents a considerable technical hurdle in realizing “zero-loss” CPV systems.

The results reported here establish a simple one-step wet-etching process that produces graded-index nanoporous ARCs on borosilicate glass (BK7 and its derivatives) surfaces featuring near-zero reflections across a broad wavelength range and for a wide angle of incidence. Additionally, the methods are cost-effective and environmentally friendly as they are based on low temperature (87 °C) neutral-solution leaching processes. Although strong acids and alkalis have long been used in corrosion of glass^23,24^, they typically generate macroporous structures that cause strong scattering of incident light, thereby preventing the formation of highly transmissive ARCs. By contrast, the neutral process reported here, which was originally formulated for use in creating durable ARCs for high energy laser optics^25,26^, is particularly well suited for formation of ARCs in the CPV system because it generates pores with diameters (~20 nm) sufficiently small to prevent light scattering and can be controlled precisely to yield highly effective ARCs. In addition, this immersive wet-etching process, compared with other deposition approaches, can be applied to surfaces with nearly any geometry, including those with demanding curvatures and hidden features. Measured transmission through flat-plate glass surfaces processed in this manner are found to achieve an average of ~99% over a broad spectral band between 380 nm and 1.3 μm and over an angular range up to ±40°, applicable to the most demanding concentrating optics used in CPV systems. More complicated surfaces of different optical elements including plano-convex lenses, ball lenses, Fresnel lenses and plano-convex lens arrays present similar values for the improvements realized in transmission. CPV systems based on microscale, triple junction (3J) solar cells provide demonstration vehicles to illustrate the functional performance of the etched surfaces in two-stage concentrating systems comprising primary plano-convex lenses together with secondary ball lenses. The etched surfaces of these types of two-stage optical concentrating systems enhance the net transmission by 13.8% and the corresponding photocurrents by up to 14.3%. Two other CPV systems that exploit optics with unconventional form factors (i.e., a Fresnel lens and a microlens array) are also examined, showing consistent photocurrent enhancements (i.e., 7.71% and 11.1%, respectively) regardless of the complexity of their surface geometries.

## Results and Discussion

**Figure 1:**
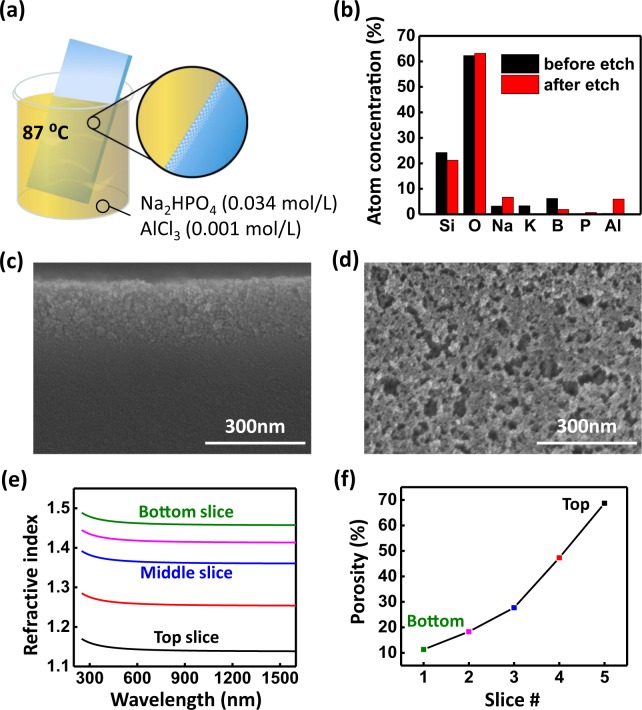
(**a**) Illustration showing the etching process. (**b**) Atomic composition of the BK7 surface as mapped by XPS before and after the etching. (**c**) Cross-sectional and (**d**) top-view SEM images of etched BK7 glass. (**e**) Modeled refractive indices of the etched BK7 glass using ellipsometry data, the etched layer is equally divided into five slices in the model, showing a gradient index profile. (**f**) Calculated porosity of 5 slices based on effective medium approximation.

The studies reported here use borosilicate crown glass. This low-thermal-expansion material is widely used for laboratory equipment, lighting, electronics and high-precision optics due to its substantial chemical, mechanical, and heat resistances. One of its most extensively applied commercial glass types (BK7) roughly contains 68% silica (SiO_2_), 15% boron oxide (B_2_O_3_), 5% potassium oxide (K_2_O), and 6% sodium oxide (Na_2_O) by weight^27–29^, with exact values varying between different manufacturers. The chemistry of etching procedures for this material has been described elsewhere^26^. Figure 1(a) schematically illustrates the one-step etching process developed in the present work in which the glass sample, after being cleaned (by acetone, isopropyl alcohol and deionized (DI) water), is immersed into a solution containing disodium hydrogen phosphate (Na_2_HPO_4_) (0.034 mol/L) and aluminum chloride (AlCl_3_) (0.001 mol/L) at a temperature of 87 °C for 26 hours. After the reaction, the sample is retrieved and cleaned by rinsing with DI water. Figure 1(b) provides the results of an X-ray photoelectron spectroscopy (XPS) study of the atomic compositions of various relevant elements on the glass surface before and after the etching process. Elements of K and B are selectively removed during the process, while relative proportions of Na and Al increase within the etched layer (angle-resolved XPS data additionally show that the proportion of Na is highest at the top of the etched surface, and decreases with depth into the bulk, while the proportion of B exhibits an opposite gradient; see Fig. S1 in the Supporting Information). The changes seen here in chemical composition support a well-elaborated theory of glass corrosion^23,30^ that divides the etching process into three distinct reactions, i.e., hydrolysis, ion-exchange and network reconstruction. Hydrolysis is initiated by hydration of the Si-O-Si network and proceeds with the release of soluble Si(OH)_4_. This leaves a broken network together with voids that allow for deeper penetration of water molecules and etchant ions. Ion-exchange occurs when alkalis (Na, K, etc.) and other network modifiers in the glass are replaced by smaller-sized protons, resulting in voids and more hydrated –Si-OH that may undergo further hydrolysis and deeper network breakdown. The hydrated –Si-OH can also reconstruct by condensation to reform Si-O-Si network elements, or modified by intermediate cations such as Al. The net result yields a graded porous network with wider channels or bigger voids closer to the surface, and with narrower channels or smaller voids deeper into the bulk of the glass. This graded-index profile is validated by the cross-sectional scanning electron microscopy (SEM) image given in Fig. 1(c), which shows a porous layer that extends ~200 nm deep into the bulk of the glass, with a graded porosity that decreases from the top-most section into the bulk. The SEM image in Fig. 1(d) captures the structures with randomly distributed nanopores from the top-down view. The average diameter of the pores at the top-most surface is estimated to be ~11 nm by statistical image processing (ImageJ), which is far below the values (>50 nm) that could lead to significant light scattering due to a transition from Rayleigh to Mie scattering^14^. Surface topography assessed by atomic force microscopy (AFM) shows distributed surface structures with feature sizes ranging from 20– 30 nm, while a pore size distribution afforded by environmental ellipsometric porosimetry (EEP) predicts an average pore diameter of 10 nm, with a tight bell-shape distribution spanning from 4 nm to 20 nm. (See Figs S2 and S3, respectively, in the Supporting Information) The surface characterization techniques (SEM, AFM and EEP) all confirm that the etched surface falls into the mesoporous regime (2–50 nm).

Optical properties of the etched surface are examined by spectroscopic ellipsometry. The ellipsometry data are analyzed based on a graded layer model that equally divides the etched surface into several numbers (N) of slices. Each slice is treated as a homogeneous medium that comprises an effective refractive index (*n*_eff_) dependent on how porous the medium is. The entire graded layer is a stack of N slices of which refractive index changes slightly in each slice. When N = 1, where the ~200 nm-thick etched surface is interpreted as being a homogenous layer, the mean squared error (MSE) is found to be 120, suggesting that the modelling is not accurate. The MSE decreases drastically when N increases, and converges to an MSE of ~9 after N > 4 (see Fig. S4 in the Supporting Information). Figure 1(e) presents estimated values of refractive indices of the etched surface modeled as five equally divided slices (N = 5; each slice is 40 nm thick) over a spectral band relevant to operation of commercial 3J solar cells from 380 nm to 1.3 µm. The bottom slice that is adjacent to the bulk of the glass has an average refractive index of 1.46 at 632.8 nm, which is slightly lower than that of bulk BK7 (*n* = 1.52). On moving the slice closer to the top of the etched surface, its refractive index decreases in a manner expected for a graded-index profile. The top slice has the lowest refractive index, 1.14, which is very close to that of the air (*n* = 1). This index gradient is sufficient to allow the reflections from the etched surface to be markedly suppressed in consequence of a better index matching. Figure 1(f) presents the calculated porosities for each slice based on an effective medium approximation (EMA) employing the Bruggeman model^13,31^:

$${f}_{glass}\frac{{n}_{glass}^{2}-{n}_{eff}^{2}}{{n}_{glass}^{2}+2{n}_{eff}^{2}}+{f}_{air}\frac{{n}_{air}^{2}-{n}_{eff}^{2}}{{n}_{air}^{2}+2{n}_{eff}^{2}}=0$$

where *n*_glass_, *n*_air_ and *n*_eff_ are the refractive indices of the glass, the air and the porous layer, respectively. The volume fraction of air (*f*_air_), i.e. the porosity, is related to the volume fraction of glass (*f*_glass_) by *f*_air_ = 1 − *f*_glass_. The topmost layer of the surface is found to consist of a layer with a porosity of up to 70%, while the bottom layer presents with a porosity of only 10%. This etched surface spans a wide modulation of porosity thought to be hard to engineer in hard materials. We note that this modulation can be easily controlled in a suitable manner by varying the solution concentration, the etching time, the temperature, and other conditions.

**Figure 2:**
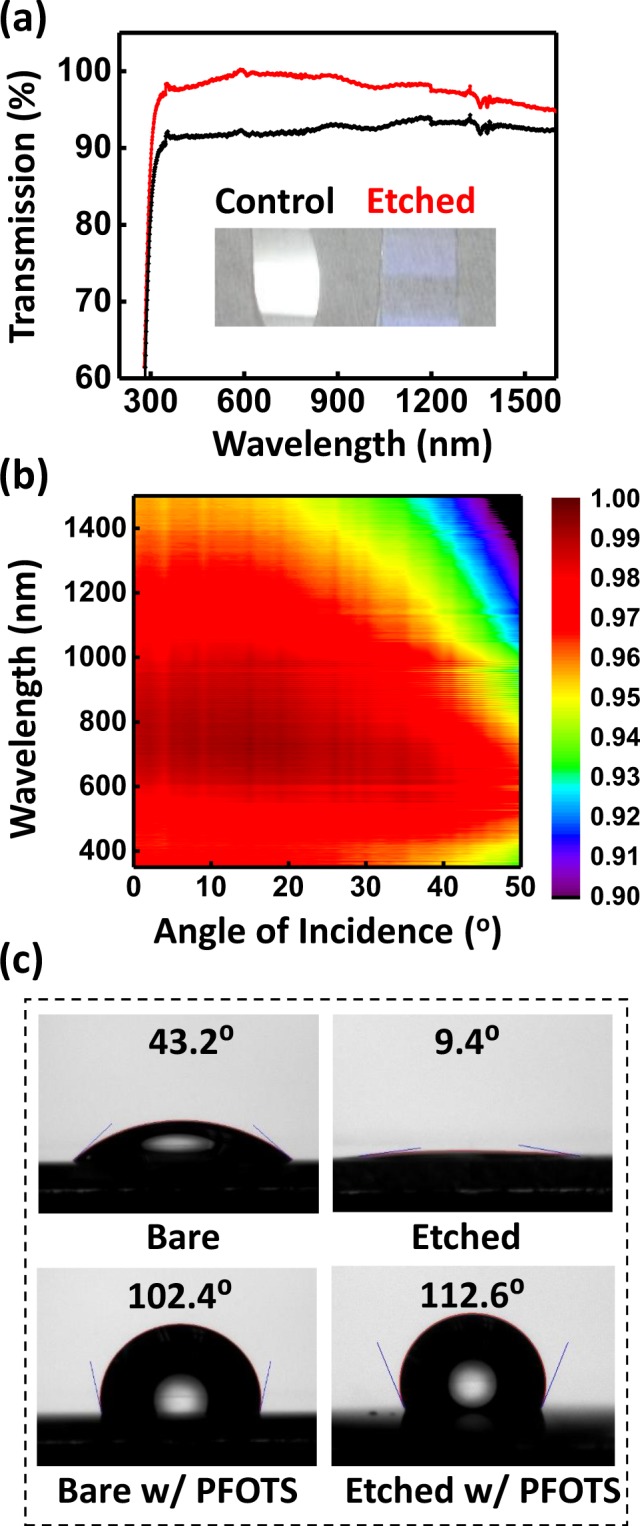
(**a**) Transmission of a flat BK7 sample before (black) and after (red) the etching. (**b**) Angular transmission data of the etched BK7 sample. (**c**) Contact angles of both bare and etched flat BK7 glass samples before and after being treated with a hydrophobic flurosilane.

A coating that comprises a gradient transition of refractive index from one medium to another is understood to display a superior AR property because the incident ray “meets no optical discontinuity”^32^. Experimental progress, however, lags far behind the theory due to the difficulty in engineering dense materials to obtain a large enough index modulation to meet the demanding requirements for such a coating. Figure 2(a) displays measured normal-incidence transmission spectra of a planar BK7 glass slide (0.5 mm thick) etched on both sides (red) and a control without the etching treatment (black). A transmittance over 99% for a wide spectrum covering the visible and near IR regions (470–940 nm) is observed, showing a nearly 9% relative enhancement over the bare control sample (~91% transmission). An average transmittance of 98.7% is obtained over an extended wavelength band from 380 nm to 1.3 µm. It is worth noting that the antireflection performance begins to fall off beyond the near IR band. This likely reflects the fact that electromagnetic fields of much longer wavelengths do not vary significantly inside the layer, experiencing in effect an abrupt interface rather than a graded one^33–35^. This broadband near-zero transmission is highly reproducible—different batches only differ in their shifted optimum peaks, likely due to their different time and temperature controls (see Fig. S5 in the Supporting Information). The graded index profile also presents omnidirectional characteristics that are difficult to achieve with homogenous coatings, which could be ascribed to the random distributions of the nanopores^14,17,36^. Figure 2(b) provides a 2D contour plot of the transmittance of the etched flat-plate glass slide as a function of the wavelength and the angle of incidence—high transmission efficiencies of over 97% are still observed at a large incident angle of 40° for the entire band (440–800 nm) of unpolarized light.

Dust, soil and water droplets deposited on the surface of the CPV optics would obstruct the optical path and considerably decrease transmission; therefore, the use of a self-cleaning coating is especially beneficial to the operation of highly efficient CPV systems. Self-cleaning surfaces are generally based on either hydrophilic or hydrophobic coatings that render extreme wetting behaviors to reduce contamination. Since surface wettability is closely related to surface roughness, the etched surfaces, with a high degree of roughness, naturally exhibit a highly wetting tendency. The surface contact angle of DI water drops from 43.2° before the etching to 9.4° after the etching, as shown in Fig. 2(c). Although a highly hydrophilic surface could protect the surface from fogging, condensed water may fill micropores, change the optical properties of the etched surface and obscure lenses. For these reasons, a highly hydrophobic surface is more desirable for optical applications involving mesoporous structures. This can be achieved, for example, by coating the optic with silane self-assembled monolayers (SAMs). Figure 2(c) shows the contact angle of DI water on bare and etched glass surfaces after treatment with trichloro(1H, 1H, 2H, 2H-perfluorooctyl)silane (PFOTS) (Coating of SAMs has a negligible impact on thin-film transmission, see Fig. S6 in the Supporting Information). The results reveal that the etched surface, though highly hydrophilic, can be readily transformed to a hydrophobic one with conventional treatments. Although most optical surfaces in CPV systems are enclosed in the module housing and isolated from the external environment, thus minimizing chances of undesirable mechanical damages, the outer surfaces of CPV optics deployed in field still suffer from mechanically-induced scratches and wear. All-glass optics would provide excellent durability to scratches and other undesirable surface damages. Unlike additive deposition methods, the etching approach offers an integral surface monolithic to the glass material that is much less susceptible to delamination upon scratching (see Fig. S7 for in the Supporting Information for mechanical tests made on the etched glass surface as compared with a polymer-based ARC). A long-term outdoor test, which is beyond the scope of this paper, may be needed to validate its resistance to mechanical damages in field deployment.

**Figure 3:**
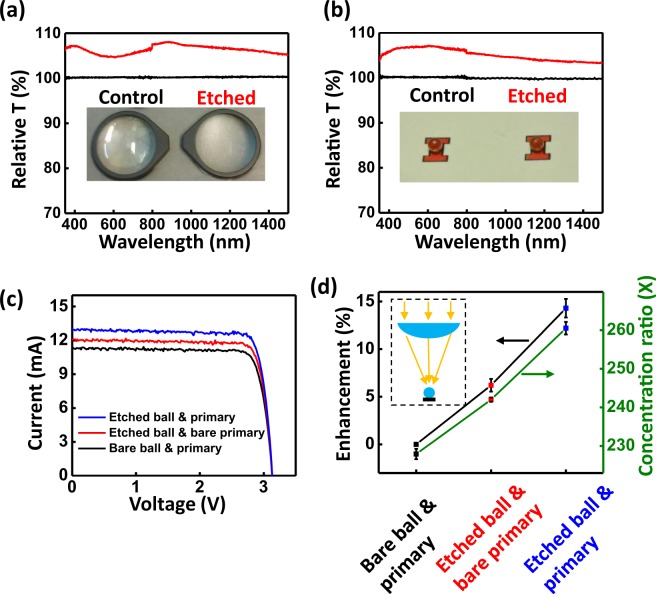
Transmission data of both (**a**) plano-convex and (**b**) ball lenses before (black) and after (red) the etching; (**c**) I–V curves and (**d**) efficiency enhancements of a 3J cell using two-stage BK7 optics before and after the etching.

The etched glass surface distinguishes itself from other ARCs in its versatile compatibility with optical elements of different form factors. This is most pronounced in the CPV optics where highly curved surfaces needed for aggressive concentrations are ubiquitous. Figure 3(a,b) provide relative transmittances of two lenses associated with the CPV optics, one being a plano-convex lens (Dia. 30.0 mm, f = 50.0 mm) and another being a ball lens (2.0 mm diameter). Transmittance enhancements in both lenses are over 6% over the wavelengths from 380 nm to 1.3 µm, which is very close to that measured in the planar flat-plate samples, with small deviations that are attributable to source-related compositional differences of the BK7 glasses. To further validate in use in complex optical systems, a two-stage optical system is assembled, with a plano-convex lens acting as the primary optical element, and the ball lens as a secondary optical element. The ball lens is bonded to a 3J (InGaP/GaAs/InGaAsNSb, 1.9 eV/1.4 eV/1.0 eV) microcell with an index-matched adhesive (*n*~1.5). Figure 3(c) captures the current (I) – voltage (V) performance of the 3 J microcell under a simulated solar spectrum before and after implement of the etched two-stage optics (for an outdoor field test, see Fig. S8 in the Supporting Information). The configuration of the CPV setup is presented schematically in the inset of Fig. 3(d). The short-circuit current (*J*_sc_) increases from 11.3 mA with unmodified optical elements, to 12.1 mA with an etched ball lens. When both etched plano-convex lens and ball lens are used for the CPV optics, the *J*_sc_ further increases to 13.0 mA. Figure 3(d) details the concentration ratios and enhancements of *J*_sc_ at various stages of ARC implements from at least four repeated measurements (including errors both from the light source and the mechanical alignment system). The etched ball surface brings a 6.2% relative enhancement, while the etched plano-convex lens brings an additional 7.6% relative enhancement. A total photocurrent enhancement of 14.3% is recorded when all the three glass/air interfaces are etched, a value resembling a total elimination of the glass/air Fresnel reflections (assuming a 4.2% loss from each interface, a total elimination of the glass/air Fresnel reflections corresponds to an enhancement of 13.7%). This implies that the reflections from the etched CPV optical concentrating systems are considerably mitigated, thereby achieving nearly zero-loss CPV platforms. All the performance characteristics measured for this CPV optic are summarized in Table 1.

**Table 1:** I–V characteristics measured under a two-stage CPV optical system.

	V_oc_ (V)	J_sc_ (mA)	FF	Power (mW)	Concentration ratio (X)	Enhancements (%)
	Representative single-point measurement				Averaged from over 4 measurements	
Bare ball & primary	3.14	11.33	0.836	29.7	227.9 ± 1.3	—
Etched ball & bare primary	3.14	12.07	0.840	31.8	242.0 ± 0.5	6.2 ± 0.7
Etched ball & primary	3.14	13.02	0.845	34.5	260.5 ± 1.6	14.3 ± 1.0

**Figure 4:**
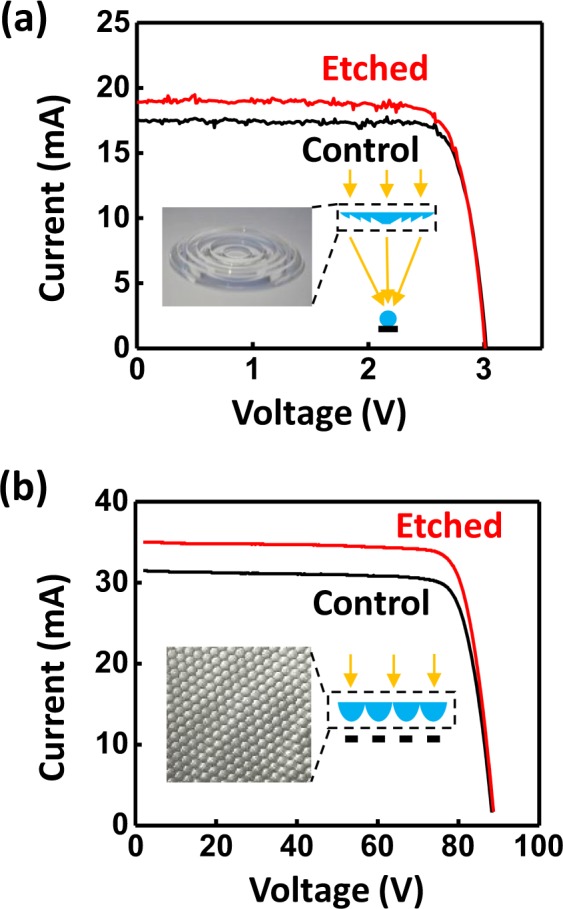
I–V data of both (**a**) a Fresnel lens and (**b**) a mirolens array couple to 3J cells before (black) and after (red) the etching.

Start-of-the-art CPV technology that employ optics with unconventional form factors, such as those with hidden surfaces, complicate processing by conventional AR coating methods. This is well illustrated for the case of Fresnel lenses, where grooves and ridges serve as self-shading structures hindering a uniform material distribution via vapor phase deposition processes. To test the current method, a Fresnel lens (Dia. 52 mm, f = 42 mm) was used instead of a plano-convex lens to construct the two-stage optics, as shown in the cartoon of Fig. 4(a). After the etching modification on the Fresnel lens, the *J*_sc_ of the 3J cell increased by 7.69%, from 17.55 mA as a bare control to 18.90 mA with the etched ARC as illustrated by the data presented in Fig. 4(a). This enhancement closely matches the one obtained from the etched plano-convex lens (7.64%), showing that the etching process well accommodates optical elements with different geometries. The inset of Fig. 4(b) shows a somewhat more complicated surface topology, a section of a low profile (~100 mm × 100 mm × 2.5 mm) hexagonal outward-facing plano-convex microlens array with a pitch of 3 mm and a focal length of 1 mm. When stacked onto a corresponding array of microscale 3J cells (18X), the construct yields a complete single-stage low-profile module configured for portable and space applications^3^. Etched surfaces formed on both sides of the microlens array significantly boost its concentrating power (with a 6.2% increase in transmission averaged from 380 nm to 1.4 µm; see Fig. S9 in the Supporting Information). Figure 4(b) shows the IV performance of the integrated module before (control) and after etching (etched). The array, which consists of 1020 microcells and an equivalent number of microlenses, sees an 11.1% enhancement in *J*_sc_ (from 31.5 mA to 35.0 mA) and a corresponding logarithmic increase in *V*_oc_ from 88.7 V to 89.0 V (such a high level of *V*_oc_, unlike the ones previously shown in the two-stage optical system, is attributed to the integration of a large number of microcells). It is worth noting that the increase in *J*_sc_, which seems to exceed what’s possible by mere elimination of Fresnel loss (~9%), also owes its contribution from the limiting subcell that benefits most from the narrower spectral band that has a higher-than-average enhanced transmission. The combined effects of *J*_sc_ and *V*_oc_ leads to a total 11.9% enhancement in efficiency (from 28.6% to 32.0%). When taken together, these results show significant opportunities exist to apply selective wet-chemical-etching processes to usefully modify the surfaces of topologically complex optical elements. The electrical performances of exemplary optics of this form are summarized in Table 2.

**Table 2:** I–V characteristics measured under a Fresnel lens and a microlens array.

	V_oc_ (V)	J_sc_ (mA)	FF	Power (W)	η
**Fresnel lens**					
No ARC	3.02	17.55	0.835	0.0442	—
ARC	3.01	18.90	0.815	0.0463	—
Δ	—	7.69%	—	4.73%	—
**microlens array**					
No ARC	88.7	31.5	0.813	2.27	28.6%
ARC	89.0	35.0	0.817	2.55	32.0%
Δ	—	11.1%	—	12.1%	11.9%

## Conclusion

A one-step etching process is demonstrated to produce nanoporous etched glass surfaces featuring a graded-index profile, self-cleaning properties, mechanical stability and supreme optical characteristics. A~99% average transmission efficiency over a broad solar spectrum (from 380 nm to 1.3 µm) and wide angles of incidence (up to ±40°) can be achieved not only for flat-plate glasses, but for optical elements with curved surfaces, ranging from plano-convex lenses and highly curved ball lenses, to ones with unconventional form factors such as Fresnel lenses and microlens arrays. Controlled surface engineering renders additional attributes, such as hydrophobicity, which is highly desired for long-term field deployment. The result also supports the concept of all-glass CPV systems, in which long-term reliability is guaranteed by true glass lenses, as compared to the more prevailing, yet less durable choice of polymer-based refractive optics.

## Methods

### Glass Elements

The planar glass sample was procured from University Wafer as 100 mm BK7 wafer. The N-BK7 plano-convex lens was procured from Thorlabs. The N-BK7 ball lens was procured from Edmund Optics. The Fresnel lens and the microlens array were press-formed from B270 by Isuzu Glass.

### Etched AR surface fabrication

The borosilicate glass samples were first cleaned by acetone, isopropyl alcohol and DI water, and then mounted onto a sealed polyethylene container containing a solution of Na_2_HPO_4_ (0.034 mol/L) and AlCl_3_ (0.001 mol/L). The temperature of the reaction mixture was maintained at 87 °C for 26 hours by a circulating water bath (Isotemp 3016H, Fisher Scientific). The borosilicate glass samples were then retrieved from the reaction bath and rinsed thoroughly by DI water.

### Surface modification

The glass samples (both etched and unetched) were cleaned thoroughly before mounted into a vacuum chamber. A few drops of trichloro (1H, 1H, 2H, 2H-perfluorooctyl) silane (PFOTS) were added to the chamber, followed by a brief decrease in chamber pressure, which enables evaporation of PFOTS. The glass samples were then retrieved after 2 hours of treatment and ready for characterization.

### Surface characterization

Surface elemental composition was characterized by XPS (Kratos Axis ULTRA) assisted with a processing software package (CasaXPS). SEM images of the etched surfaces were obtained on Helios 600i and JEOL 7000F Scanning Electron Microscopes. Normal incidence spectral transmittance curves were measured by a spectrometer (Varian Cary 5G). Absolute transmission appeared in Fig. 2(a) and S6 was obtained with air as a reference while relative transmission appeared in Fig. 3(a,b) and S9 was obtained using a bare control optical element as a reference. The angle-resolved transmission spectra, refractive index and thickness of the etched surface were obtained by RC2 DI and focused RC2 XI spectroscopic ellipsometers (J. A. Woollam Co.). The EEP measurements were performed using a J.A. Woollam Co. environment cell along with the M-2000 DI spectroscopic ellipsometer. Contact angles against DI water were measured by using a goniometer/tensiometer (ramé-hart model 250).

### Electrical characterization

The ball lens was bonded to the 3J cell with Norland Optical Adhesive 61 (NOA 61). I-V performance of the microscale 3J cell was measured with a Keithley 2400 sourcemeter. Oriel 91192-1000W Solar Simulator with an AM1.5G filter (calibrated to one sun, 100 mW·cm^−2^) was used as the illumination source. The optics positioned by an x-y-z precision manipulator to ensure proper optical alignment.

## Electronic supplementary material

Supplementary Information
