# Supplementary material for: Solution processes for ultrabroadband and omnidirectional graded-index glass lenses with near-zero reflectivity in high concentration photovoltaics
Source: Sci Rep. 2018 Oct 8;8:14907. doi: 10.1038/s41598-018-33200-9 (PMC6175897; doi:10.1038/s41598-018-33200-9)
Supplement: Supplementary file 1 — Supplementary Information [file 41598_2018_33200_MOESM1_ESM.docx]

Supporting Information

Solution processes for ultrabroadband and omnidirectional graded-index glass lenses with near-zero reflectivity in high concentration photovoltaics

Junwen He,† Yuan Yao,† Kyu-Tae Lee,†,* Nina Hong, Brent Fisher, Rabab R. Bahabry, Jung Woo Lee, Jeonghyun Kim, Seungyong Han, Sanjay V. Kalidindi, Jae-Hwan Kim, Sung Bong Kim, Jaewon Choi, Hongwoo Jang, Myeong Namkoong, Scott Burroughs, Muhammad Hussain, Ralph G. Nuzzo,* and John A. Rogers*


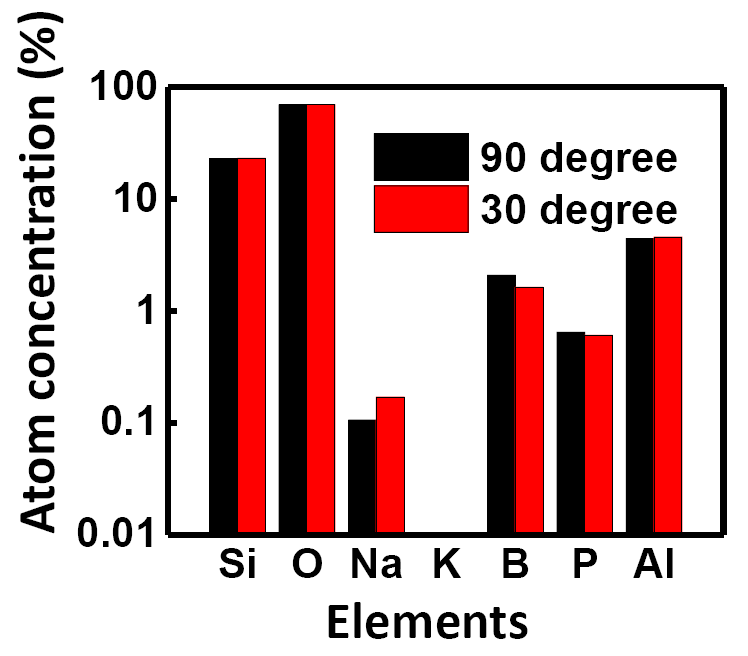


**Figure S1.** Angle-resolved XPS data showing that the proportion of Na is highest at the top of the etched surface, and decreases along the depth into the bulk, while the proportion of B presents an opposite gradient.


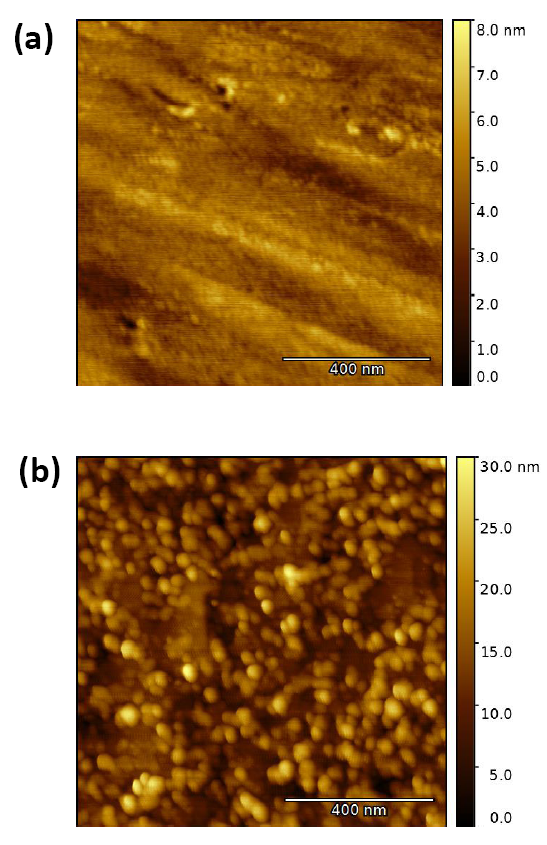


**Figure S2.** Atomic force microscopy images of the glass surfaces (a) before and (b) after applying the wet-etching process.


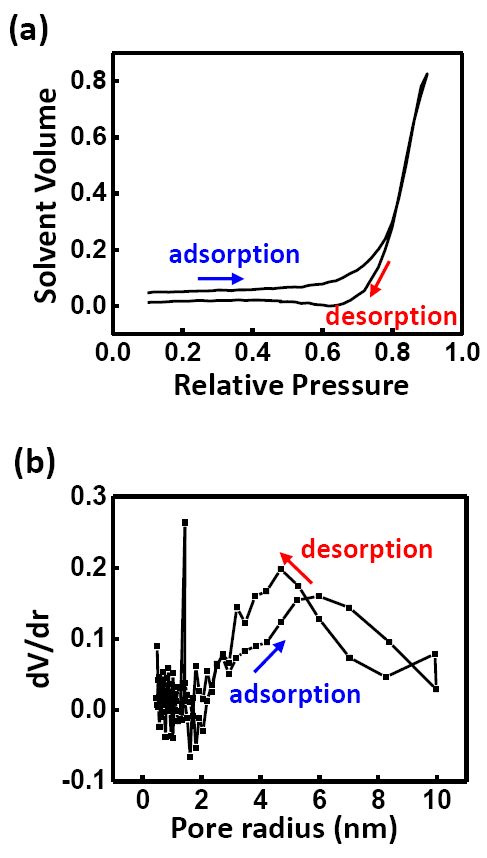


**Figure S3.** Pore size calculations based on environmental ellipsometric porosimetry. (a) Adsorption and desorption of water by the etched porous surface at controlled pressure. (b) Calculated pore size distribution assuming cylindrical pores. The porosity calculated by the EMA theory is compared with an experimental value determined by EEP. As the graded porosity makes the porosimetry analysis non-ideal, only the top slice refractive index is traced at each relative pressure. The maximum solvent volume is close to 80%, a value agreeing reasonably well with the EMA based porosity. The pore size distribution for the top slice of the etched BK7 glass is calculated by the derivative of the condensed solvent volume in the pores vs. relative pressure.


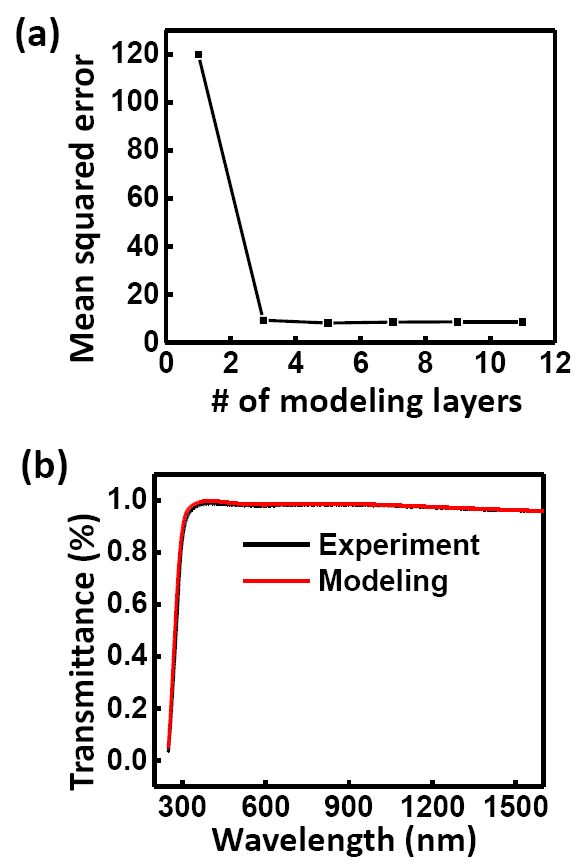


**Figure S4.** (a) Mean squared error (MSE) values depending on the number of the modeling layers. (b) Fitting results of spectroscopic ellipsometry data based on 5 modeling layers.

Figure S5. Batch-to-batch variation of the etched planar glass samples. Near zero transmission across broadband is highly reproducible, while different batches only differ in their shifted optimum peaks.


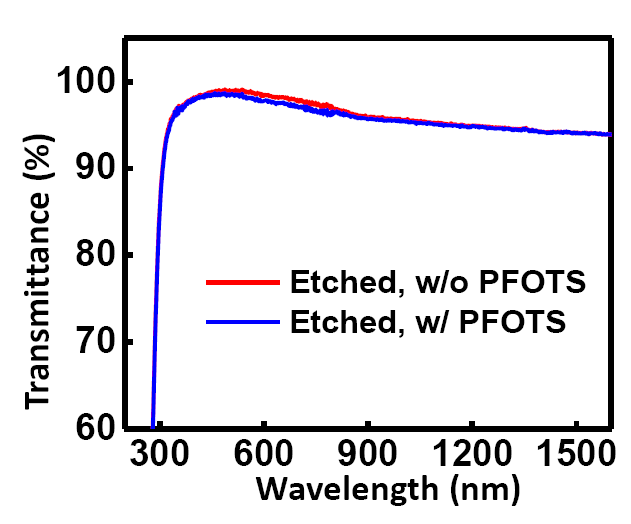


**Figure S6.** Measured transmission spectra before (red) and after (blue) the hydrophobic treatment.


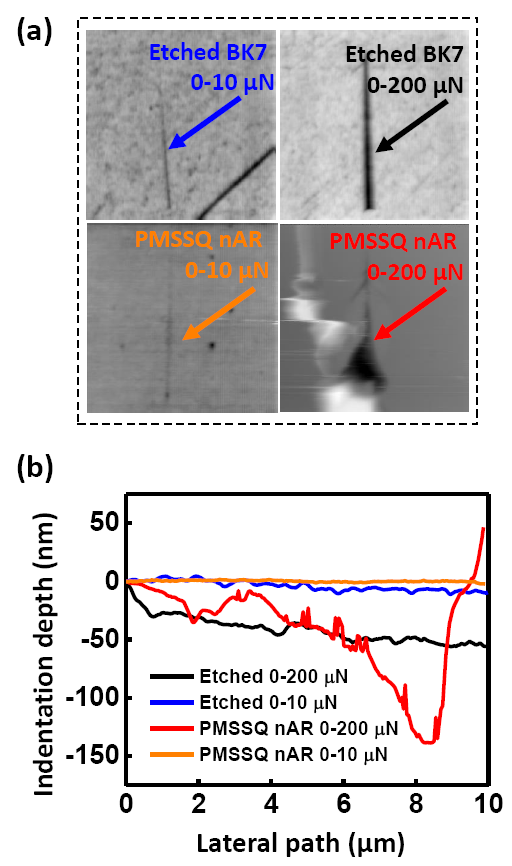


**Figure S7.** (a) Scanned topological maps of both a etched glass surface and a porous PMMSQ coating after a line scan with a nanoindenter; (b) Indentation depth extracted from the nanoindenter measurements: the coated ploymer film (index~1.2) exhibits slightly better scratch resistance under a low indentation force (0-10 µN), however, under a high force (0-200 µN), the polymer film starts to delaminate from the surface.


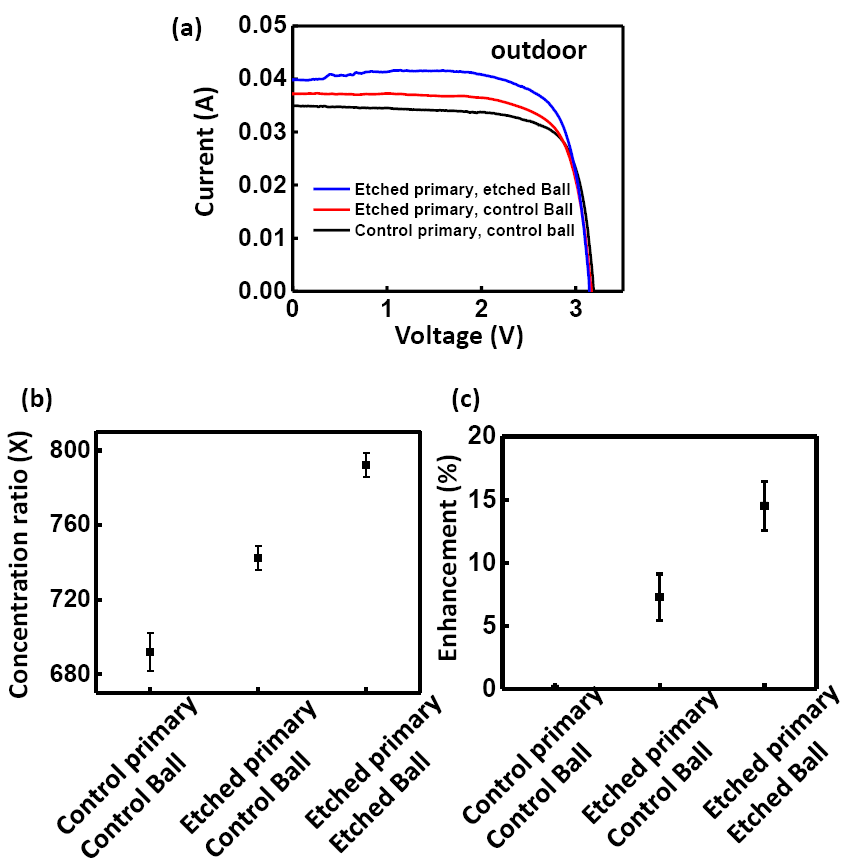


**Figure S8.** Outdoor PV measurements for the two-stage CPV optics with incremental glass treatments. Unsteady IV curves and bigger error bars are due to unstable weather/irradiance conditions during data gathering.


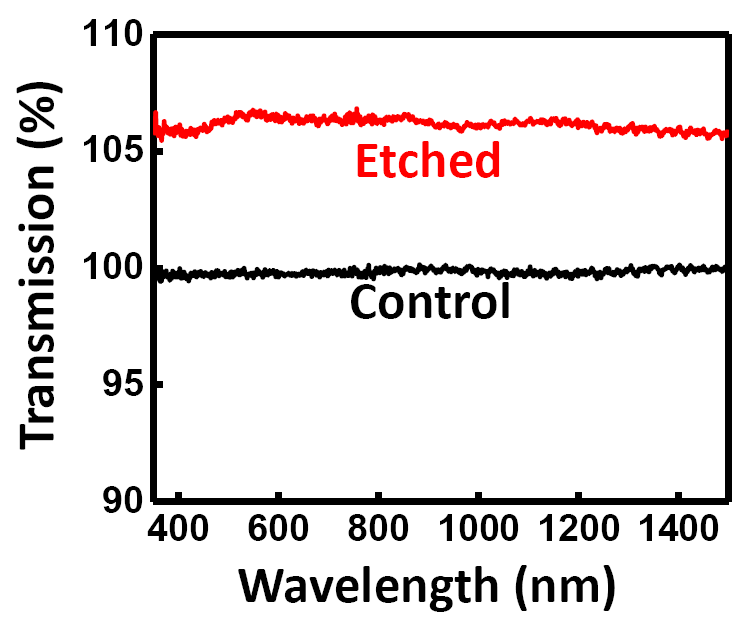


**Figure S9.** Relative transmission spectra of etched surfaces formed on both sides of the microlens array presenting 6.2% of transmission improvement, averaged from 380 nm to 1.4 µm.
